# Improved Cordycepin Production by *Cordyceps militaris* KYL05 Using Casein Hydrolysate in Submerged Conditions

**DOI:** 10.3390/biom9090461

**Published:** 2019-09-07

**Authors:** Soo Kweon Lee, Ju Hun Lee, Hyeong Ryeol Kim, Youngsang Chun, Ja Hyun Lee, Hah Young Yoo, Chulhwan Park, Seung Wook Kim

**Affiliations:** 1 Department of Chemical and Biological Engineering, Korea University, 145, Anam-Ro, Seongbuk-Gu, Seoul 02841, Korea (S.K.L.) (J.H.L.) (H.R.K.) (J.H.L.); 2 Department of Interdisciplinary Bio-Micro System Technology, College of Engineering, Korea University, 145 Anam-Ro 5, Seongbuk-Gu, Seoul 02841, Korea; 3 Department of Food Science and Engineering, Dongyang Mirae University, 445, Gyeongin-Ro, Guro-Gu, Seoul 08221, Korea; 4 Department of Biotechnology, Sangmyung University, 20, Hongjimun 2-Gil, Jongno-Gu, Seoul 03016, Korea; 5 Department of Chemical Engineering, Kwangwoon University, 20, Kwangwoon-Ro, Nowon-Gu, Seoul 01897, Korea

**Keywords:** casein hydrolysate (CH), cordycepin, *Cordyceps militaris*, submerged culture

## Abstract

Cordycepin, a beneficial bioactive product specifically found in *Cordyceps*, has received attention in various bioindustrial applications such as in pharmaceuticals, functional foods, and cosmetics, due to its significant functions. However, low productivity of cordycepin is a barrier to commercialization. In this study, *Cordyceps militaris* was mutated by UV irradiation to improve the cordycepin production. The highest producer KYL05 strain was finally selected and its cordycepin production was increased about 1.5-fold compared to wild type. In addition, the effects of culture conditions were fundamentally investigated. Optimal conditions were as follows: pH 6, temperature of 25 °C, shaking speed of 150 rpm, and culture time of 6 days. Effects of medium component on cordycepin production were also investigated by using various carbon and nitrogen sources. It was found that glucose and casein hydrolysate (CH) were most effective as carbon and nitrogen sources in cordycepin production (2.3-fold improvement) with maximum cordycepin production of about 445 mg/L. In particular, production was significantly affected by CH. These results should be of value in improving the efficiency of mass production of cordycepin.

## 1. Introduction

*Cordyceps militaris*, widely used as a traditional medicinal mushroom due to various biological activities of the body since ancient times in Asia, is a fungus belonging to the family Clavicipitaceae and a species of *Cordyceps* genus [1]. In general, *Cordyceps* has unique behaviors such as winter-insect and summer-plant form. Their spores can enter into a specific living insect and kill the host by feeding. Their hyphae can grow from inside of the host. They can pass the winter inside the host, eventually forming fruiting bodies on the surface of host insect’s cadaver in the summer [2]. The *Cordyceps* genus includes over 500 species, among them, it has been one of the most famous functional mushrooms in traditional Chinese medicines [3]. Wild fruiting bodies of *Cordyceps* are very expensive because specific hosts and conditions are required for the growth, and it is difficult to find in the nature.

Cordycepin (3′-deoxyadenosine), one of nucleoside analogues, was first isolated from the medicinal mushroom *Cordyceps militaris*. The difference between cordycepin and adenosine is the lack of the 3′-hydroxyl group in cordycepin and not the “3′ position of the ribose part” [3,4]. Various functions of cordycepin have been reported such as immunomodulatory, antioxidant, anticancer, anti-inflammatory, and antimicrobial activities [5,6]. Therefore, cordycepin has received attention due to its potential application in functional food and healthcare fields.

Cordycepin is commonly known to be produced mainly in *C. militaris*, but the amount of cordycepin produced per unit dry weight is reported to be very small (about 0.5%). Since cultured *C. militaris* contains various components, it is necessary to perform many steps of purification to remove non-target ingredients to obtain pure cordycepin [7]. In addition, a large amount of *C. militaris* is required for commercial production of cordycepin. Thus, artificial cultivation of wild *Cordyceps* at solid state with various insect pupae and larvae have been studied for commercial use [6]. Especially, nutritional requirements, environmental conditions and inoculum preparation were investigated for the cultivation. However, the solid culture of mushrooms has a disadvantage that it takes long time to complete a fruiting body development [8]. Submerged mycelial culture could be an alternative solution to overcome the low productivity. Previously, several studies were reported to obtain cellular or extracellular substances in the submerged culture of *C. militaris* since it has various advantages such as higher mycelial production, cultured in compact area, shorter culture time, and easy contamination control (cultured in closed system) [8,9,10,11].

The roduction of secondary metabolites from microorganisms, including fungi, is known to require specific amino acids, precursors, inducers, or elicitors [12]. The components of culture medium can also significantly affect the production yield. Carbon and nitrogen sources play an important role because these components are directly related to cell growth and metabolic biosynthesis. In addition, there have been reports on the use of low-cost biomass for economical production of high value-added materials (biorefinery) in bioindustry [13,14,15,16,17,18]. However, the study of the effects of nutritional requirement of *C. militaris* on cordycepin synthesis is still in the basic stages of research. Reports on effective media for cordycepin production are limited.

Therefore, the major objective of this study was to improve cordycepin production in submerged culture. Among the various *C. militaris*, a strain with high yields of cordycepin production per cell mass was selected, and the culture conditions such as temperature, pH, and shaking speed were fundamentally investigated. In addition, a medium composed of different carbon and nitrogen sources was prepared and the effective composition for cordycepin production was determined based on experimental results. Investigation of secondary metabolite production through changes in medium components requires a number of trials. It is possible to understand more clear and simple correlations by the experiments. Thus, a fundamental study of *C. militaris* culture under submerged conditions would be beneficial for scale-up in industrial processes.

## 2. Experimental

### 2.1. Microorganisms

*C. militaris* KCTC6064 and KCTC6862 were purchased from the Korea collection for type cultures (Jeongeup-si, Jeollabuk-do, Korea). *C. militaris* KYL05 was mutated from wild type *C. militaris* KCTC6064 by ultraviolet irradiation. An about 1% survival rate was obtained after 5 min. The colonies that appeared on the plates were isolated, and a mutant of *C. militaris*, strain KYL05, was finally selected by an agar-diffusion method. The strain was inoculated on a potato dextrose agar (PDA; composition, potato starch 4 g/L, glucose 20 g/L, and agar 15 g/L) plate at 25 °C for 7 days [19,20].

### 2.2. Media and Culture Conditions

#### 2.2.1. Seed Culture of *C. Militaris*

The inoculum of seed culture was performed by adding 0.5% Tween 80 in PDA slant agar medium, and then 4% solution (about 2.0 × 10^9^ spores/mL) was transferred into the broth seed medium. The compositions of seed medium were as follows: potato dextrose broth (PDB; 4 g/L potato starch and 20 g/L glucose). The conditions of seed culture were performed at 25 °C for 3 days in shaking incubator (200 rpm) with a 250 mL Erlenmeyer flask containing 50 mL of broth seed medium [21].

#### 2.2.2. Main Culture of *C. Militaris*

The compositions of main medium were as follows: YPD (pH 6, 10 g/L yeast extract, 10 g/L peptone, 20 g/L glucose), 0.1 g/L KH_2_PO_4_, 0.2 g/L K_2_HPO_4_∙3H_2_O, and 0.2 g/L MgSO_4_7H_2_O. The seed broth of *C. militaris* KYL05 was transferred into the main medium about 4%, and the cultivation was performed at 25 °C for 6 days in a shaking incubator (200 rpm) in a 250 mL Erlenmeyer flask containing 50 mL of broth main medium [22].

#### 2.2.3. Effects of Temperature, Initial pH, and Shaking Speed

The effect of initial temperature on cell growth and cordycepin production by *C. militaris* KYL05 was carried out at various temperature of 15, 25, 30, and 37 °C for 6 days in a shaking incubator (Multi Shaking Incubator/BF-150SIR-2R, BioFree, Seoul, Korea) in a 250 mL Erlenmeyer flask containing 50 mL of broth main medium. The effect of initial pH on cell growth and cordycepin production was performed in a 250 mL Erlenmeyer flask containing 50 mL of broth main medium with the different initial pH of 4, 5, 6, 7, 8, 9, and 10. The cultivation was performed at 25 °C for 6 days in a shaking incubator (200 rpm). The effect of shaking speed on cell growth and cordycepin production was carried out at 25 °C for 6 days in a shaking incubator with different rotatory shaking speeds of 100, 150, and 200 rpm [22,23].

#### 2.2.4. Effects of Carbon Source and Nitrogen Source

The effect of carbon sources on the cordycepin production by *C. militaris* KYL05 was performed at 25 °C for 6 days in shaking incubator (150 rpm) in a 250 mL Erlenmeyer flask containing 50 mL of experimental medium. The component of experimental medium is 5 g/L yeast extract and 5 g/L peptone with 20 g/L different carbon source (glucose, galactose, sucrose, fructose, lactose, mannose, cellulose, and carboxymethyl cellulose (CMC)) at pH 6. In addition, the effect of nitrogen sources on the cordycepin production was performed under the same conditions as the experimental method for the effect of the carbon source. The experimental medium for nitrogen source was consisted of 20 g/L glucose with 8 different nitrogen sources of 20 g/L (yeast extract, soytone, tryptone, malt extract, CH, bacto peptone, proteose peptone, and whey). All experiments were performed at least in triplicate to ensure reproducibility. Experimental data are presented as mean ± standard deviation of triplicate measurements.

### 2.3. Analytical Methods

The cell growth was monitored at every 24 h sampling by measurement of the dry cell weight (DCW). The cultural broth was centrifuged at 8000 *g* for 30 min at 4 °C, and then the sediment was washed by distilled water. The DCW was measured by the weight of samples through a pre-weighed filter paper (Whatman GF/C, Maidstone, UK) and dried in a vacuum oven (VO-20X, Jeiotech, Daejeon, Korea) for 48 h at 50 °C.

The cordycepin concentration was determined by high performance liquid chromatography (HPLC) system equipped with a 260 nm diode array detector (Primaide 1430, Hitachi, Japan). X-Bridge C18 column (5.0 μm, 4.6 mm × 250 mm, Waters, Milford Massachusetts, USA) was used for the analysis at room temperature, and the mobile phase in the column was 15% (*v*/*v*) methanol in 20 mM phosphoric acid at a flow rate of 1.0 mL/min.

Analysis of amino acids was carried out by using amino acid analyzer (Hitachi L-8900, Hitachi, Japan) attached Hitachi HPLC packed column with ion-exchanging resin no. 2622 PF (4.6 mm × 60 mm) and UV detector (VIS1: 570 nm, VIS2: 440 nm). In this study, Wako L-8500 buffer solutions PF-1, 2, 3, 4, and RG were used. The injection volume was 20 μL and the determination of sample was performed using Ninhydrin Reagent Set (Wako Chemical Inc, Osaka, Japan). All samples were run in triplicates.

## 3. Results and Discussion

### 3.1. Microbial Strain Selection for Cordycepin Production

**Figure 1:**
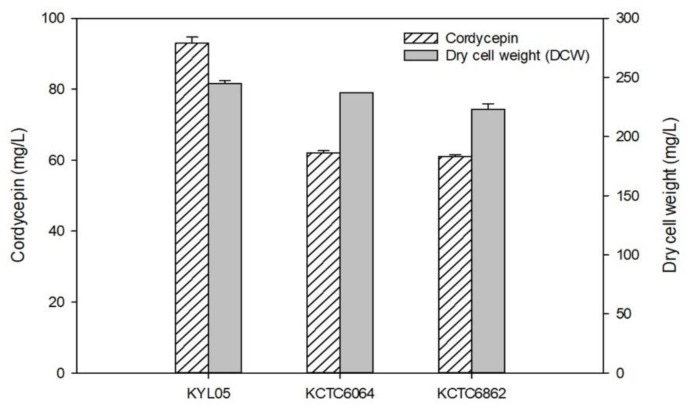
Cordycepin production and dry cell weight from *C. militaris* by three strains.

To produce cordycepin known to be produced only in *Cordyceps*, it is essential to select a strain with high productivity. Based on two strains (*C. militaris* KCTC 6064 and KCTC 6862) known to have excellent productivity for cordycepin, ultraviolet irradiation was performed to induce random mutagenesis, and the strain (KYL05) with the highest productivity was selected by an agar-diffusion method. Figure 1 shows cordycepin concentration, dry cell weight (DCW), and cordycepin productivity at 6-day culture for three strains of *C. militaris*. Mycelial growth was similar for KCTC 6064 (237 mg/L), KCTC 6862 (223 mg/L) and KYL05 (245 mg/L) strains. The highest cordycepin concentration was achieved by KYL05 strain (about 93 mg/L) under the same conditions, and the concentration by KCTC 6064 and KCTC 6862 strain were found to be 62 and 61 mg/L, respectively. Cordycepin productivity (mg-cordycepin/g-cell) was calculated based on the cell mass per cordycepin production and the results shows that the productivity by strain KCTC 6064, KCTC 6862 and KYL05 was about 265, 275, and 380 mg/g-cell, respectively. Therefore, *C. militaris* KYL05 that produced the highest productivity for cordycepin was finally selected in the current study.

### 3.2. Determination of Culture Conditions for Cordycepin Production

**Figure 2:**
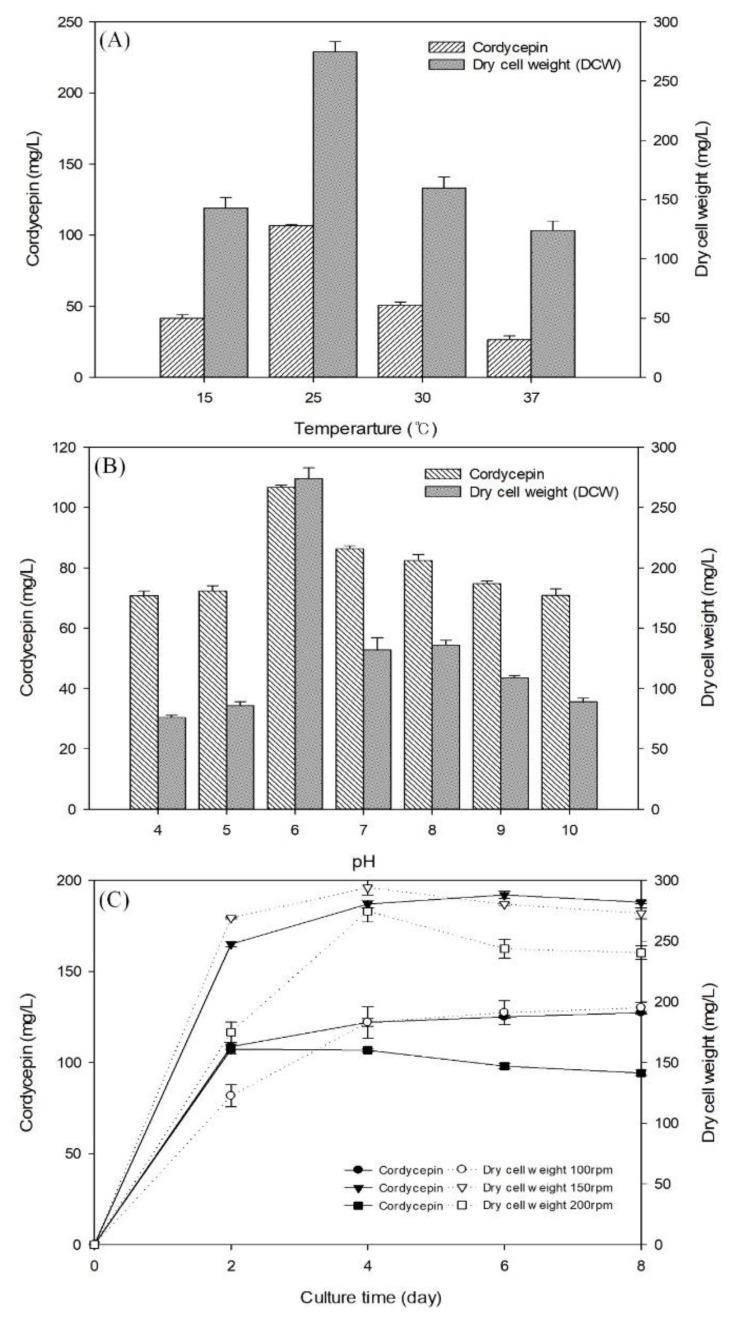
Effects of temperature, initial pH, and various shaking speeds on the cordycepin concentration and dry cell weight by *C. militaris* KYL05. (**A**) Different temperatures of 15 °C (○), 25 °C (▽), 30 °C (□), and 37 °C (◇); (**B**) initial pH of basal medium; and (**C**) different shaking speeds of 100 (●, ○), 150 (▼, ▽), and 200 (■, □) rpm were used. Error bars in the figure indicate the standard derivations from three independent samples.

Growth rate and metabolite production from fungi are known to be significantly affected by culture conditions such temperature, pH and agitation speed [24,25]. Here, effects of initial temperature (15–37 °C), pH (4–10), and shaking speed (100–200 rpm) on cordycepin production by *C. militaris* KYL05 and cell growth were investigated. Figure 2A shows cordycepin production and cell growth at different temperatures for 4 days. The maximum cordycepin production and cell growth were achieved at 25 °C (107 and 275 mg/L, respectively). Average cordycepin production and cell mass at 25 °C were found to be 2.6-fold and 2.2-fold higher than those at other temperatures, respectively. This result shows that 25 °C is the optimum temperature for cordycepin production. Subsequent cultures of *C. militaris* KYL05 were all incubated at 25 °C. Other reports have suggested that the highest level of cordycepin is produced at 25 °C for various *Cordyceps* strains. Results of our study were found to be in accordance with previous reports [25]. In general, initial pH of culture medium can significantly influence cellular morphology and metabolite biosynthesis since it can affect solubility of salts, ionic state of substrates, cell membrane function, and metabolite biosynthesis [26]. Effects of initial pH on cordycepin production and cell growth are shown in Figure 2B. Results showed that the maximum cordycepin production and cell growth were achieved at pH 6 (112 and 278 mg/L, respectively). Thus, an initial medium pH of 6 was used for further applications. Agitation is an important factor for mass (substrate and oxygen) and heat transfer. Morphological change of fungi can occur due to shear stress [8,24]. Figure 2C shows the culture profiling of *C. militaris* KYL05 for cordycepin production and cell mass at different shaking speeds. The maximum cordycepin production was achieved by using shaking speed of 150 rpm, leading to cordycepin production level of 192 mg/L after 6 days of culture. In addition, the highest cell growth of about 290 mg/L was achieved by 150 rpm at 4 days. These results indicated that shaking speed of 150 rpm was the most effective one for both cordycepin production and cell growth compared to other speeds. Therefore, optimal culture conditions of *C. militaris* KYL05 were determined to be YPD medium at pH 6, temperature of 25 °C, shaking speed of 150 rpm, and culture duration for 6 days.

### 3.3. Effect of Carbon and Nitrogen Source on Cordycepin Production

**Figure 3:**
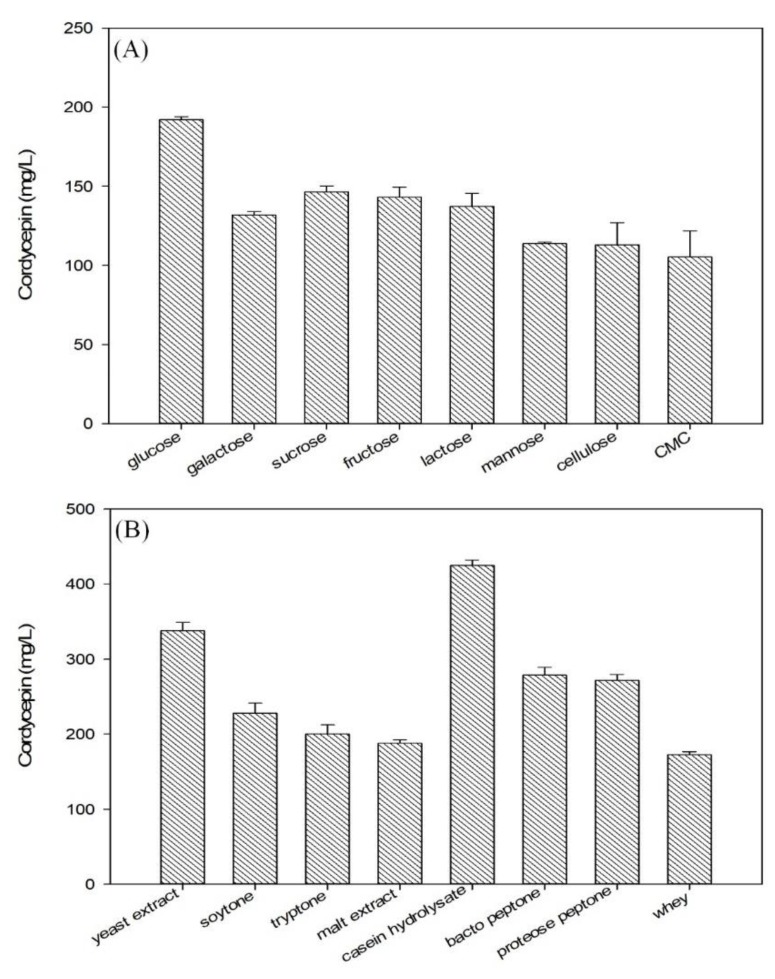
Effects of various (**A**) carbon sources (glucose, galactose, sucrose, fructose, lactose, mannose, cellulose, and CMC) and (**B**) nitrogen sources (yeast extract, soytone, tryptone, malt extract, CH, bacto peptone, proteose peptone, and whey) on cordycepin production by *C. militaris* KYL05. The culture was performed at determined conditions (pH 6 at 25 °C and 150 rpm for 6 days).

Previous reports have indicated that types of carbon and nitrogen sources play an important role in cell growth and metabolites (primary and/or secondary metabolites) production in the fermentation process of fungal cells [27,28,29,30,31]. The effect of nutrient sources on cordycepin production by *C. militaris* KYL05 was investigated by using 8 different types of carbon sources and nitrogen, respectively. Figure 3A shows effects of carbon sources on cordycepin production at the previously determined conditions. As a result, glucose was found to be the most effective one for cordycepin production. Thus, glucose was selected as a suitable carbon source for further production process. Similarly, effects of nitrogen sources on cordycepin production were investigated under the same conditions. Results are shown in Figure 3B. It was confirmed that the production of cordycepin was significantly dependent on the type of nitrogen source. The maximum cordycepin production was achieved at about 445 mg/L by using CH. Therefore, CH was finally selected as a beneficial nitrogen source in the current study.

**Figure 4:**
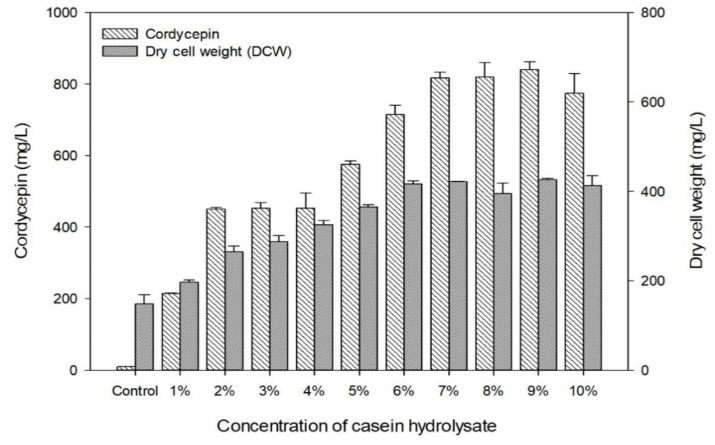
Effects of CH concentrations on dry cell weight and cordycepin production by *C. militaris* KYL05 in a submerged cultivation. Culture was carried out at pH 6, 25 °C, and 150 rpm for 6 days.

In addition, the effect of CH concentration on cordycepin production and cell growth were investigated since the concentration of nitrogen source can have a significant effect on the cell growth and cordycepin production. Experiments of cultivation profiling were carried out in the basal medium (2% glucose) supplemented with 1%–10% CH. Figure 4 shows results of cordycepin production and cell growth under different concentrations of CH. As control, the basal medium (2% glucose) was not supplemented with CH. In this result, the production of cordycepin was increased as the concentration of CH increased. The maximum production of 840 mg/L was achieved at 9% CH-supplemented medium. The cell growth was continuously increased to 6% CH concentration, however, there was no significant effect on the cell growth in 6% CH-supplemented medium. As a result, cordycepin production increased as the concentration of CH increased in supplemented medium. However, cordycepin productivity which was calculated based on g-dry cell weight per g-cordycepin production was not correlated with concentration of CH. Cordycepin productivities in 2% and 9% CH-supplemented media were about 0.58 and 0.51 g-cordycepin/g-dry cell weight, respectively. In the experiments, white crystals were observed when the medium was supplemented with CH at concentration higher than 2% supplemented medium. This phenomenon is presumed to be due to addition of solute above its solubility in the medium. In particular, the solubility of CH in water is 2% according to the manufacturer, indicating that casein was precipitated due to addition above its solubility. Higher than 2% CH in supplemented medium might be expected to cause various problems as follows: low productivity, low economic efficiency due to the use of excess CH, incompatibility of fermentation due to crystallization of CH, and others. Finally, 2% was determined to be the most suitable concentration of CH for cordycepin production by *C. militaris* KYL05.

A number of independent studies, including the present study, have sought to improve culture conditions for the production of cordycepin. These results are summarized in Table 1. Although, various *C. militaris* strains were used, reported culture conditions were similar: temperature of 24–25 °C, pH of 4.8–6.5, agitation speed of 100–200 rpm, and culture time of 5–21 days. The effect of culture medium on cordycepin production was investigated. The most beneficial carbon and nitrogen sources were also determined, respectively. In all reports, glucose was chosen as the most suitable carbon source. Its concentration was determined to be 1%–4%. In most studies, the most suitable nitrogen source was determined as peptone at about 1% concentration in the medium [9,32,33,34]. In our study, CH was determined to be the most effective nitrogen source. The production of cordycepin was found to be 445 mg/L at optimum conditions. The maximum cordycepin production was reported by Fan et al. (2012) and about 597 mg/L cordycepin was achieved by 4% glucose and 1% peptone medium at 25 °C with 110 rpm at 20-day culture [9]. The productivity was calculated to be about 30 mg/L∙day, which calculated the production of cordycepin per day. The cordycepin production was the highest, however, the productivity was slightly lower because the culture time was longer than 20 days. It shows that the maximum productivity (74.2 mg/L∙day) was investigated as our result obtained by the relatively short culture time of 6 days. Wen et al. (2017) reported that cordycepin production of 3005.83 mg/L was achieved by 2% sucrose and 2% peptone at 25 °C in a liquid static culture for 40 days [35]. Compared to submerged culture, liquid static culture can confirm a significantly higher cordycepin concentration, however, the disadvantage is very long cultivation times of more than a month. Cordycepin productivity by static culture was calculated to be 75.1 mg/L∙day and the productivity was not significantly different from our results. The main difference between submerged and static cultures is the application of agitation during fermentation (liquid static culture techniques are not stirred).

In this study, a significant improvement in cordycepin productivity is achieved by utilization of CH. Until now, there has been no reports on the application of CH as an optimal source for cordycepin production in submerged cultures of *C. militaris*. However, CH has been used as a major source for producing other secondary metabolites. In a previous study, CH has been reported to be a significant source for accumulating ginseng saponin and cell growth during *Panax ginseng* cell cultures. Wu et al. have reported that the volumetric saponin yield by feeding sucrose (30 g/L) with CH (0.5 g/L) increased about 3.5-fold (1131 mg/L) compared with the control group (251 mg/L) [36]. These reports suggest that CH has affected the production of secondary metabolites. Thus, it can be assumed that certain components in CH were involved in the production of fungal secondary metabolites. Wang et al. have reported that the amino acid composition of glutamate was abundant in CH [37]. In addition, preliminary assay on a *Cardamine pratensis* L. cell suspension has confirmed the positive effect of glutamine, the most important amino acid contained in CH [38].

Oh et al. have reported that *C. militaris* can enrich bioactive compounds associated with nucleotides, carbohydrates, and amino acid metabolism during fruit body development. They confirmed that the biosynthesis of cordycepin might be regulated by glutamine and glutamate pathway based on the patterns of amino acid metabolism [12]. In particular, Leung and Wu have reported effects of glutamine and glutamate feeding (10–30 mmol/L) on mycelium growth and cordycepin production of *C. sinensis*-HK1 in shake-flask culture. Cordycepin production by glutamine and glutamate feeding was increased about 2-fold than in the control group [39]. In addition, studies on the effect of amino acids on metabolic regulatory mechanisms, such as cordycepin synthesis, have recently been reported [40,41]. Therefore, it can be presumed that CH is most effective in the cordycepin production in comparison with other organic nitrogen sources because glutamate is abundant in CH. To demonstrate this hypothesis, we analyzed the amino acid components contained in three nitrogen sources (CH, yeast extract, and peptone) that were effective in cordycepin production, and the results are shown in Table 2. Each nitrogen source contains various amino acids and their composition was different. In particular, the content of glutamate was analyzed to be about 18%, 12.4%, and 10% of CH, yeast extract, and casein, respectively, and it can be seen that CH contains the highest glutamate. These results provide evidence to support our hypothesis and can be related to increased cordycepin production and glutamic acid content in nitrogen sources. However, because of the various of amino acids included in CH and that the mechanism of cordycepin synthesis has not yet been fully understood, this conclusion may be premature, and further studies on cordycepin synthesis are required.

This study could provide useful information for the development of economic and efficient production of cordycepin. In particular, in the bioindustry where fermentation processes such as functional foods, pharmaceuticals and cosmetics are used, the method of adding specific ingredients to the medium could be applied to improve the production yield.

## 4. Conclusions

In this study, the effects of culture conditions such as temperature, pH, and shaking speed on mycelial growth of *C. militaris* KYL05 were investigated under submerged conditions to improve cordycepin production. In addition, the effect of medium components (various carbon and nitrogen sources) on cordycepin production was investigated under the previously determined conditions. Among various carbon sources, glucose was found to be the most suitable for cordycepin. The maximum production (~450 mg/L) of cordycepin was achieved when the medium was supplemented with 2% CH. Following the reports, amino acid composition of glutamate was abundant in CH and the biosynthesis of cordycepin could be regulated by glutamate pathways based on amino acid metabolism patterns. Therefore, the abundance of the nucleotide precursor glutamate in CH may impact cordycepin biosynthesis by promoting nucleotide synthesis. Fundamental information obtained from this study could be used in the development of process using *C. militaris* for large scale cordycepin production.

## Figures and Tables

**Table 1:** Summary of culture conditions of *C. militaris* for cordycepin production.

Strain	Culture Conditions				Carbon Source (g/L)	Nitrogen Source (g/L)	Cordycepin Production (mg/L)	Cordycepin Productivity (mg/L∙day)	Ref.
	Temp. (°C)	RPM	pH	Time (day)					
*C. militaris*	25	110	6	20	Glucose 40	Peptone 10	597	29.9	[9]
*C. militaris*	25	110	4.8	18	Glucose 42	Peptone 16	245	13.6	[30]
*C. militaris*	25	110	5	17	Glucose 10	Peptone 10	346	20.4	[32]
*C. militaris* KCTC6862	24	120	6.5	5	Glucose 10	Peptone 10	22	4.4	[33]
*C. militaris* KCTC16932	24	120	6.5	5	Glucose 10	Peptone 10	23	4.6	
*C. militaris* DGUM32003	24	120	6.5	5	Glucose 10	Peptone 10	39	7.8	
*C. militaris*	25	200	4.8	17	Glucose 40	Peptone 10	201	11.8	[34]
*C. militaris* KYL05	25	150	6	6	Glucose 20	CH 20	445	74.2	This study

**Table 2:** Amino acid composition in nitrogen sources.

Amino Acid	Amino Acid Concentration (g-Amino Acid/100 g-Nitrogen Source)		
	CH	Yeast Extract	Peptone
Aspartic acid	6.12	6.54	5.82
Threonine	3.69	3.19	2.18
Serine	4.42	3.29	3.33
Glutamate	18.01	12.44	9.97
Proline	7.48	2.80	10.13
Glycine	1.69	3.07	20.00
Alanine	2.75	5.75	8.87
Valine	5.58	4.16	2.88
Isoleucine	4.33	3.45	1.76
Leucine	7.46	4.92	3.48
Tyrosine	3.19	0.70	0.48
Phenylalanine	3.88	2.90	2.15
Lysine	6.65	5.29	3.89
Histidine	2.11	1.38	0.82
Arginine	2.84	3.41	7.10
Cysteine	0.47	1.07	0.34
Methionine	2.46	1.08	0.84
Total content	83.13	65.42	84.06
